# Newly Identified Essential Amino Acids Affecting *Chlorella ellipsoidea* DGAT1 Function Revealed by Site-Directed Mutagenesis

**DOI:** 10.3390/ijms19113462

**Published:** 2018-11-04

**Authors:** Baocheng Sun, Xuejie Guo, Chengming Fan, Yuhong Chen, Jingqiao Wang, Zanmin Hu

**Affiliations:** 1 State Key Laboratory of Plant Cell and Chromosome Engineering, Institute of Genetics and Developmental Biology, Chinese Academy of Sciences, Beijing 100101, China; bcsun@genetics.ac.cn (B.S.); xuejie.guo01@gmail.com (X.G.); yhchen@genetics.ac.cn (Y.C.); 2 College of Life Sciences, University of Chinese Academy of Sciences, Beijing 100049, China; 3 Institute of Economical Crops, Yunnan Agricultural Academy, Kunming 65023, China; jingqiaowang25@gmail.com

**Keywords:** diacylglycerol acyltransferase, site-directed mutagenesis, heterologous expression, total fatty acid content, triacylglycerol

## Abstract

Diacylglycerol acyltransferase (DGAT) is a rate-limiting enzyme in the synthesis of triacylglycerol (TAG), the most important form of energy storage in plants. Some residues have previously been proven to be crucial for DGAT1 activity. In this study, we used site-directed mutagenesis of the *CeDGAT1* gene from *Chlorella ellipsoidea* to alter 16 amino acids to investigate effects on DGAT1 function. Of the 16 residues (L482R, E542R, Y553A, G577R, R579D, Y582R, R596D, H603D, H609D, A624R, F629R, S632A, W650R, A651R, Q658H, and P660R), we newly identified 5 (L482, R579, H603, A651, and P660) as being essential for DGAT1 function and 7 (E542, G577, R596, H609, A624, S632, and Q658) that significantly affect DGAT1 function to different degrees, as revealed by heterologous expression of the mutants in yeast strain INVSc1. Importantly, compared with *CeDGAT1*, expression of the mutant *CeDGAT1Y553A* significantly increased the total fatty acid and TAG contents of INVSc1. Comparison among CeDGAT1Y553A, GmDGAT1Y341A, AtDGAT1Y364A, BnDGAT1Y347A, and BoDGAT1Y352A, in which tyrosine at the position corresponding to the 553rd residue in CeDGAT1 is changed into alanine, indicated that the impact of changing Y to A at position 553 is specific for CeDGAT1. Overall, the results provide novel insight into the structure and function of DGAT1, as well as a mutant gene with high potential for lipid improvement in microalgae and plants.

## 1. Introduction

Triacylglycerols (TAGs) are important storage lipids that can be used for edible oil and/or be processed for biodiesel. In algae and higher plants, TAGs are mainly synthesized by diacylglycerol acyltransferase (DGAT), a rate-limiting enzyme of the TAG synthesis pathway [1]. DGAT is currently classified into four types: DGAT1, DGAT2, WS/DGAT, and cytoplasmic DGAT3 (CytoDGAT). DGAT1 and DGAT2, which are mainly associated with the endoplasmic reticulum (ER), are microsomal enzymes; although they catalyze similar biochemical reactions, their protein sequence similarity is extremely low. In general, DGAT1 has a wider role in TAG synthesis, whereas DGAT2 activity is more related to the accumulation of specific fatty acids [2]. Bifunctional WS/DGAT predominantly catalyzes the synthesis of wax esters and minor amounts of TAGs in *Arabidopsis thaliana* (WSD1) [3]. Cytoplasmic DGAT3 has been reported to be involved in wax synthesis in a variety of plants [4,5], but its specific role in TAG synthesis remains unclear.

Since the first *DGAT1* was cloned from mouse [6], many *DGAT1* genes have been isolated from various organisms [2,7,8,9,10,11,12,13,14,15,16,17]. *DGATs* vary in DNA size and structure among plants and generally contain 15–17 exons [1]. The exons of *DGAT1s* in animals are often clustered and distributed at the 3′ end of the gene, whereas plant *DGAT1* exons are distributed throughout the gene. In addition, plant DGAT1 proteins generally comprise between 480 and 550 amino acids. Structural differences of DGAT1s among species mainly exist at the *N*-terminus, which may be related to the selectivity of different plant DGAT1s for the acyl–CoA substrate [18]. DGAT1s typically contain 8–10 transmembrane domains and a hydrophilic N-terminus [9]. Although the sequences of DGAT1s from different species vary widely, some common functional regions have been recognized, including phosphorylation sites, the DAG-binding domain, a leucine zipper, and a conserved proline residue that participates in acyl CoA binding [8,10].

Given the important function of *DGAT1*, scientists have attempted to utilize the gene for crop improvement. Overexpression of *DGAT1* significantly increases the seed oil content of different plants [19,20]. For example, overexpression of *AtDGAT1* in Arabidopsis resulted in increases in seed lipids and seed weight by 11–28% and by 2.5–32.3%, respectively [19]. Additionally, expression of *TmDGAT1* in Arabidopsis and *Brassica napus* resulted in a net increase of seed oil content of 10–33% and 11–15%, respectively [21], and expression of *JcDGAT1* in Arabidopsis caused a 30–41% increase in seed oil content [15].

DGAT1 belongs to the membrane-bound O-acyltransferase (MBOAT) family [22], which includes members such as acetyl-CoA acetyltransferase (ACAT) 1, ACAT2, and Lysophosphatidic acid acyltransferase (LPAT). Overall, membrane-bound enzymes are more difficult to study than are soluble proteins, due to a lack of knowledge regarding their crystallographic three-dimensional structures. Nonetheless, site-directed mutagenesis experiments have shown that a conserved histidine residue is required for activity of human ACAT1 and ACAT2 and mouse DGAT1 [23,24,25]. Indeed, site-directed mutagenesis is a feasible approach to studying the relationship between enzyme structure and function [26,27]. The crystal structure of DGAT1 has not yet been reported to date [28] and its three-dimensional structure has only been speculated based on some protein structure prediction software. However, the structure–function relationship of DGAT1 has been studied by generating variants. It has been reported that DGAT1 activity in *Tropaeolum majus* was almost completely lost after the amino acids at positions F439 and P216 were changed and that the substitution of S197 with A in a putative (SNF)-related protein kinase 1(SnRK1) target site resulted in a strong increase in DGAT1 activity in the range of 38% to 80% [21]. Furthermore, the phenylalanine at position 469 of DGAT1 was found to affect the concentrations of seed oil and oleic acid in maize lines [29]. Additionally, *B*. *napus* DGAT1 variants at sites 441 and 447 generated by directed evolution resulted in increases in TAG contents in *Nicotiana benthamian*a leaves, indicating that these amino acid sites substitutions potentially affect DGAT1 activity [27]. Although some residues have been found to be essential for DGAT1 activity, it is necessary to understand the roles of more sites on enzyme function and to obtain more genes that, when mutated, could be used for improving oil crops.

In a previous study, we cloned a *DGAT1* gene from *Chlorella ellipsoidea* and found that *CeDGAT1* can improve oil crops by significantly increasing the lipid content by 12–18% and the 1000-seed weight by 6–29% [30]. In this study, we investigated the structure–function relationship of CeDGAT1 by site-directed mutagenesis. We found that seven amino acids are essential for CeDGAT1 function and that another eight can significantly affect CeDGAT1 function to different degrees, with one significantly increasing the total fatty acid content of yeast compared with that of the native protein. This study provides new clues for clarifying the structure–function relationship of DGAT1 and a more useful *DGAT1* gene that can be used for oil crop improvement.

## 2. Results

### 2.1. Sequence Conservation among DGAT1s from Different Species and the Selection of Mutagenesis Sites

**Figure 1:**
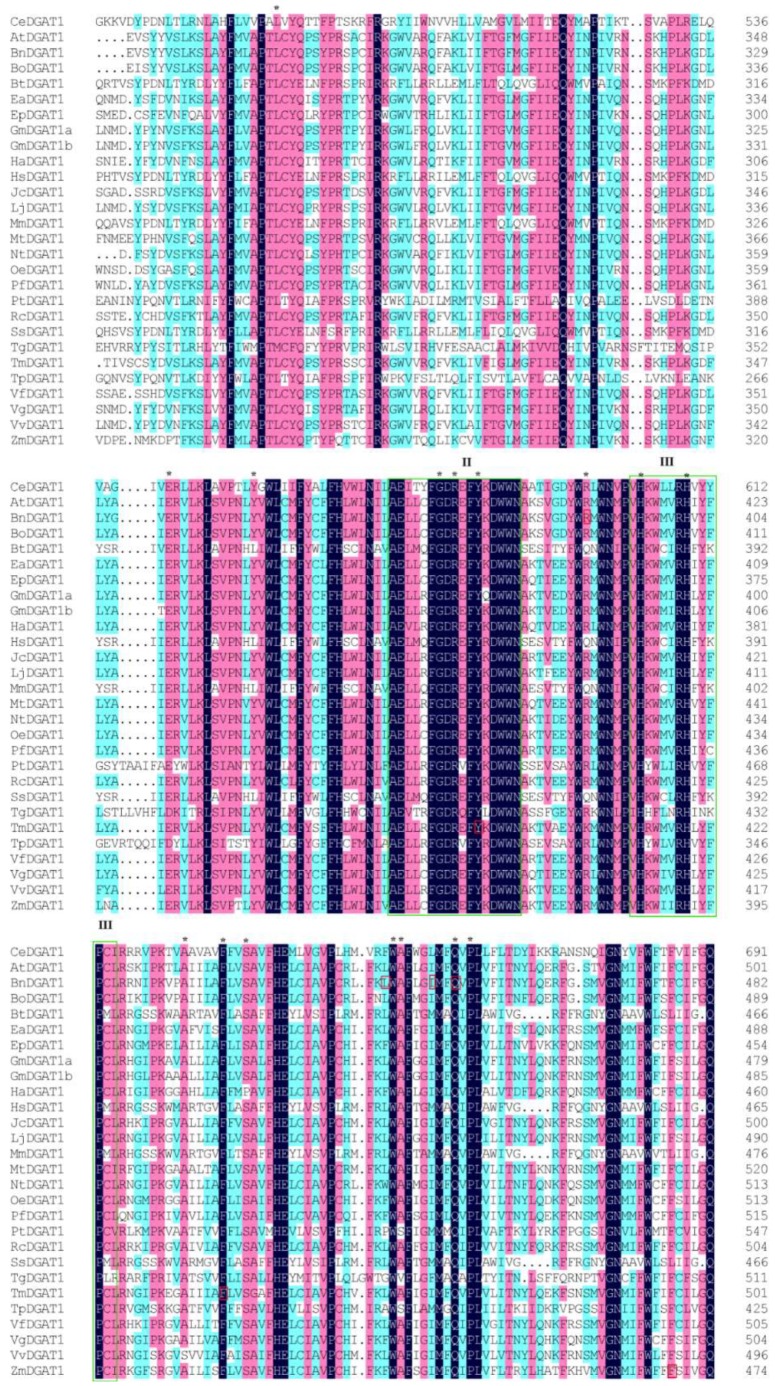
Partial multiple sequence alignment of the 28 DGAT1s from 27 species and mutagenesis site selection. The deduced amino acid sequences of these diacylglycerol acyltransferase 1 (DGAT1s) were aligned using MAFFT v6.847b [31] with the L-INS-i algorithm. Conserved amino acids are shaded in black (90–100% similarity, the highest conservation), pink (70–90% similarity, higher conservation), and light blue (50–70% similarity, high conservation). Conserved motifs (domain II, the fatty acid protein signature domain, and domain III, the DAG-binding site domain) are marked by green rectangles, Y392 and F439 in TmDGAT1; F469 in ZmDGAT1; R388, L441, I447, and Q450 in BnDGAT1 are marked by red boxes, which were previously proven to affect the activity of DGAT1 in *Tropaeolum majus*, *Zea mays*, and *Brassica napus*, respectively, and the 16 selected conserved residues mutated are labeled by asterisks. The GenBank accession numbers and sources of *DGAT1* genes are listed in Supplementary Materials Table S1.

**Figure 2:**
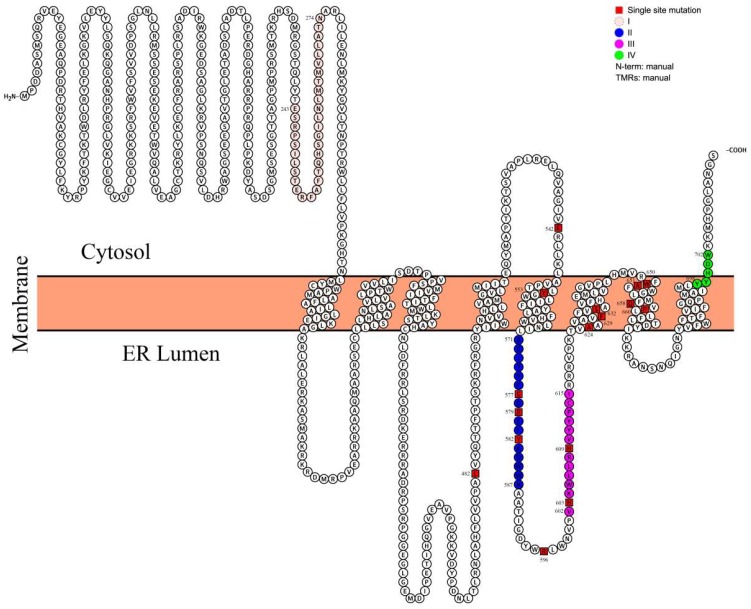
The predicted topology model of CeDGAT1. The membrane topology of CeDGAT1 was predicted by Protter [32] and TMHMM [33] software with default parameters according to the software manuals. The amino acid sequence of CeDGAT1 is represented by black circles. Single site mutations are represented by red boxes. Functional sites predicted by the Prosite database are presented by four circles with different colors: I, the acyl-CoA binding domain; II, the fatty acid protein signature domain; III, the DAG-binding site domain; and IV, the putative endoplasmic reticulum retrieval motif at the C-terminus. The number represents the position of the amino acid.

Amino acid sequence conservation among DGAT1s was compared using 28 sequences from 27 different organisms (listed in Table S1). In this multiple sequence alignment, we found that variations were mainly located at the N-terminus and that the conserved sequence comprised amino acids 233–705, including functional domains I, II, III, and IV. However, only one completely conserved amino acid (same in all 28 sequences) was found in domain IV and two in domain I (Figure S1). Figure 1 illustrates the most conserved region, including domains II and III, which are boxed. More than 100 conserved amino acid residues were identified in this partial alignment (Figure 1 from the 459th amino acid to the 691th amino acid of CeDGAT1), which are shaded in black, pink, and light blue, according to the degree of similarity (from high to low). Among these residues, 16 sites (L482, E542, Y553, G577, R579, Y582, R596, H603, H609, A624, F629, S632, W650, A651, Q658, and P660), labeled by asterisks in Figure 1, were selected for mutagenesis. These 16 sites in the full-length CeDGAT1 sequence are shown in Figure S2 and are not randomly distributed in the predicted topology model of Figure 2. G577, R579, and Y582 are located in domain II and H603 and H609 in domain III, both of which are predicted to be inside the endoplasmic reticulum (ER) lumen. L482 is found on the stem of the 4th transmembrane domain (TMD) and R596 on the loop between the 5th and the 6th TMDs, which are inside the ER; E542 was on the stem of the 5th TMD, which is outside the ER; and the other sites are all within the TMD (Figure 1). Among the selected 16 residues, only Y582, F629, R596, and Q658 have been studied previously in *Tropaeolum majus* [21] and *B*. *napus* [27].

The selected amino acids were mutated and the sites of amino acids before and after the mutation are listed in Table 1. The amino acid represented by the first letter was mutated to the amino acid represented by the second letter at the indicated position. The general principles of the mutagenesis were that the residues were mutated to a different one to alter the chemical nature of the residues (i.e., charged to noncharged or hydrophobic to hydrophilic).

### 2.2. Analysis of Mutants Function by GC–MS

**Figure 3:**
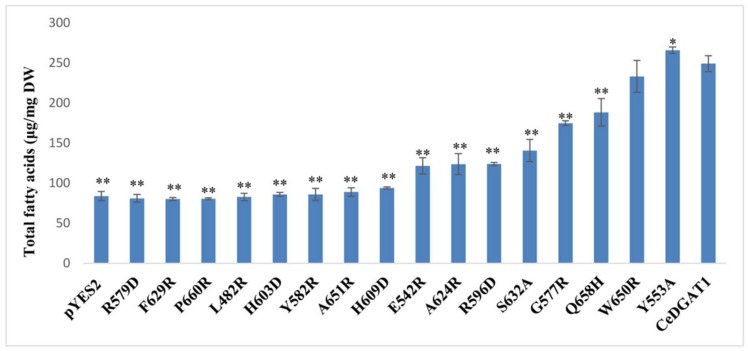
Total fatty acid content of transgenic yeast expressing different *CeDGAT1* mutant genes. pYES2, yeast INVSc1 transformed with the empty vector pYES2.0; *CeDGAT1*, yeast transformed with pYES–*CeDGAT1*. Statistical analysis was performed with Student’s *t*-test. Asterisks indicate a significant difference from INVSc1 cells expressing CeDGAT1 (* *p* < 0.05, ** *p* < 0.01). The results are expressed as the mean ± standard deviation (*n* = 3).

The mutant genes and wild-type *CeDGAT1* were inserted into the yeast expression vector pYES2.0 (Figure S3) and transformed into yeast strain INVScI; expression of *CeDGAT1* and the mutants in transgenic yeast was confirmed by RT-PCR (Figure S4). In addition, the total fatty acid contents were analyzed by gas chromatography–mass spectrometry (GC–MS), and the results are shown in Figure 3. Compared with INVScI transformed with the empty plasmid, the total fatty acid content of yeast cells carrying *CeDGAT1* was significantly increased by 197.30%, suggesting that *CeDGAT1* had a strong impact on lipid synthesis in yeast. Conversely, the total fatty acid contents of the yeast cells carrying the genes expressing the R579D, F629R, P660R, L482R, H603D, Y582R, and A651R mutants of *CeDGAT1* were similar to the level of the empty vector-harboring cells, suggesting that mutations at these seven positions caused complete loss of CeDGAT1 function. Other mutations at positions H609, E542, A624, R596, S632, G577, and Q658 resulted in increased fatty acid contents by 12.03%, 45.00%, 47.60%, 47.80%, 67.81%, 108.70%, and 124.80%, respectively, compared to empty vector-carrying cells, but decreases by 62.32%, 51.24%, 50.36%, 50.29%, 43.56%, 29.80%, and 24.39%, compared to yeast *CeDGAT1-*carrying cells. More importantly, Y553A significantly increased the total fatty acid content of yeast by 217%, approximately 6.75% higher than that of yeast expressing the *CeDGAT1* gene. These results suggest that each of these sites may play an important role in DGAT1 function. However, the mutation of W650 had no effect on the function of CeAGAT1.

### 2.3. Mutant Functional Analysis by TLC

**Figure 4:**
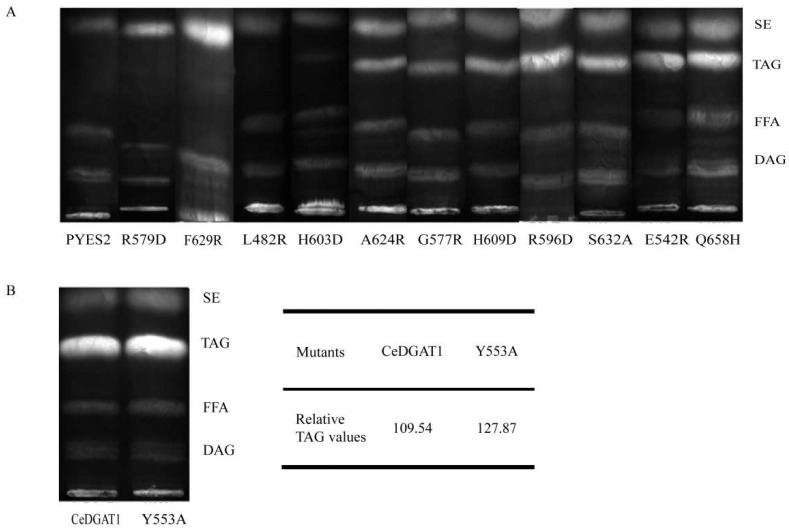
Lipid analysis by TLC of yeast H1246 cells carrying different *CeDGAT1* mutant genes. (**A**) pYES2, yeast H1246 transformed with empty pYES2.0 vector; R579D, F629R, L482R, H603D, A624R, G577R, H609D, Y582, R596D, S632A, E542R, Q658H, and Y553A, yeast H1246 cells carrying the corresponding mutant of *CeDGAT1*. (**B**) *CeDGAT1*, yeast H1246 transformed with pYES–*CeDGAT1*; Y553A, yeast H1246 cells carrying the corresponding mutant of *CeDGAT1*. The relative TAG values are evaluated by semiquantitative densitometry calculated through ImageJ 1.51w. SE, steryl ester; TAG, triacylglycerol; FFA, free fatty acid; DAG, diacylglycerol. The bands represent the existence of each product.

*CeDGAT1* and some selected *CeDGAT1* mutants causing significantly altered total fatty acid contents in yeast were separately transformed into the yeast mutant H1246, a TAG-deficient *Saccharomyces cerevisiae* quadruple mutant strain [34] lacking *DGA1*, *LRO1*, *ARE1*, and ARE2*,* and total lipid contents were analyzed by thin-layer chromatography (TLC); the results are shown in Figure 4. A band representing TAG was not found for H1246 cells expressing the mutants R579D, F629R, L482R, and H603D, indicating that the yeast cells expressing these mutants could not synthesize TAG, which was consistent with the change in total fatty acid content. By contrast, TAG production was observed with yeast cells expressing mutants A624R, G577R, H609D, R596D, S632A, E542R, Q658H, and Y553A, suggesting that these mutants can synthesize TAG. The results further demonstrate that the mutated sites R579D, F629R, L482R, and H603D are crucial for DGAT1 function.

To clearly illustrate the function of the mutant Y553A, we further compared the relative TAG production of H1246 cells expressing *CeDGAT1* and Y553A by semiquantitative densitometry calculated with ImageJ 1.51w (Figure 4B). The results indicated that the TAG production in the presence of the Y553A mutant was approximately 10% greater than that in the presence of *CeDGAT1*. This finding further indicates that the Y553A mutant significantly improved TAG synthesis in comparison to the native *CeDGAT1*.

### 2.4. Mutation of DGAT1 from Different Species Corresponding to Position 553 of CeDGAT1

**Figure 5:**
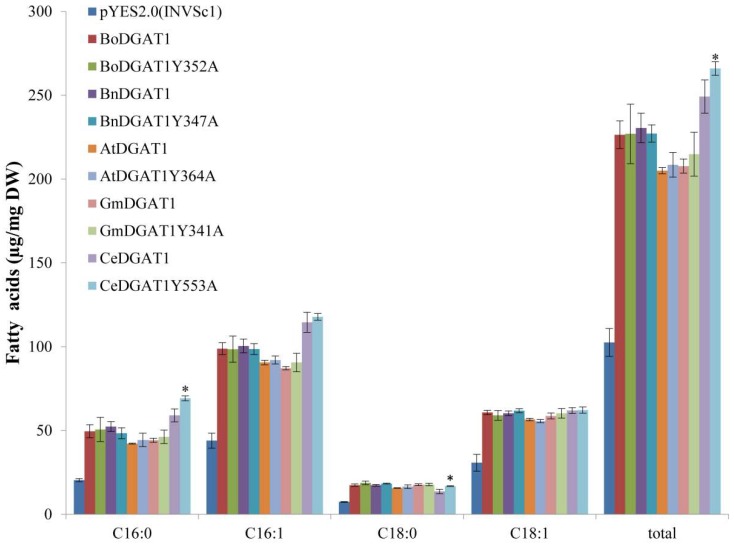
Fatty acid analysis of yeast with harboring *DGAT1* genes from different species mutated at the corresponding 553rd position of CeDGAT1. C16:0, palmitic acid; C16:1, palmitoleic acid; C18:0, stearic acid; C18:1, oleic acid; and total, the sum of the C16:0, C16:1, C18:0, and C18:1 contents. Asterisks indicate a significant difference between the mutant and native genes (* *p* < 0.05). The results are expressed as the mean ± standard deviation (*n* = 3).

Among the mutants, only the expression of *CeDGAT1Y553A* significantly increased the TAG and total fatty acid contents of yeast, compared to that of *CeDGAT1*. To examine whether the position of DGAT1 corresponding to the 553rd position in CeDGAT1 from different species has a similar effect, we cloned the *GmDGAT1*, *AtDGAT1*, *BnDGAT1*, and *BoDGAT1* cDNA sequences, predicted to have the position corresponding to 553 in CeDGAT1, and changed the Y residue to A residue through site-directed mutagenesis. The mutant genes (*CeDGAT1Y553A*, *GmDGAT1Y341A*, *AtDGAT1Y364A*, *BnDGAT1Y347A*, and *BoDGAT1Y352A* in pYES2.0), *CeDGAT1*, and the empty vector (pYES2.0) were transformed into *S. cerevisiae* INVSc1, and total fatty acid contents were measured through GC–MS. The results showed that the mutation did not affect the function of GmDGAT1, AtDGAT1, BnDGAT1or BoDGAT1 (Figure 5), suggesting that the effect of changing tyrosine to alanine at position 553 is specific for CeDGAT1.

## 3. Discussion

Analysis of the relationship between the structure and function of DGAT will be helpful for understanding the mechanism of lipid synthesis. However, detailed three-dimensional structural information on DGAT1 is not available, owing to its multiple transmembrane protein properties and purification difficulties [28]. Enzyme variants produced by single amino acid substitutions can be useful for gaining insight into structure–function relationships in the absence of a three-dimensional structure [29]. Conservation analysis and site-directed mutagenesis are indirect and direct approaches, respectively, of studying the relationship between protein structure and function by predicting and/or identifying functionally important residues in protein sequences. Previous studies have shown that domains I, II, III, and IV are essential for DGAT1 activity [14,21] and some residues have been confirmed to play an important role in the activity of this enzyme [14,27,29]. In this study, we selected 16 conserved sites located within different regions of the membrane topology structure of CeDGAT1 for directed mutagenesis. We found that seven residues (482nd, 579th, 582nd, 603rd, 629th, 651st, and 660th amino acids) are essential and that another seven residues (542nd, 577th, 596th, 609th, 624th, 632nd, and 658th amino acids) are significantly important for the function of CeDGAT1. More importantly, we found a mutation (Y553A) that significantly enhanced the function of CeDGAT1.

CeDGAT1 contains several conserved domains: Domain I, an acyl-CoA binding motif; domain II, a fatty acid-binding protein signature motif; domain III, a DAG-binding motif; and domain IV, a putative C-terminal ER retrieval motif [30]. These motifs are conserved among plant DGAT1s [14,21]. According to Protter [32] and TMHMM [33], CeDGAT1 possesses eight putative transmembrane domains, which is consistent with its role as an integral membrane protein that has been shown to be localized to the ER [2,30]. Based on the topology model (Figure 2), we further mapped the mutated single amino acid sites in the predicted three-dimensional structure of the CeDGAT1 protein (Figure S5) and found that, except for L482 and R596, these sites are either in/near functional domains or in the ER membrane. Our results showed that the mutation of G577, R579, and Y582 in domain II and H603 and H609 in domain III significantly decreased the function of CeDGAT1; based on the complete and/or almost complete loss of DGAT1 function when mutated, R579, Y582, and H603 are essential for CeDGAT1. The mutation of amino acids A624, F629, S632, A651, Q658, and P660 in different TMDs also obviously decreased the function of DGAT1, of which F629, P660, and A651 are essential. L482 in the 4th TMD stem inside the ER is essential, and E542 in the 5th TMD stem outside the ER and R596 in the loop between domains II and III play important roles in maintaining function. Furthermore, the mutation of Y553 in the ER membrane of the 5th TMD, near the fatty acid-binding protein motif, notably improved CeDGAT1 function. Overall, our results suggest that different conserved sites located in the confirmed functional domains, ER lumen, ER, or stem and loop significantly influence the function of *CeDGAT1*. Interestingly, the mutation of W650, the most conserved amino acid residue, had no effect on the function of CeDGAT1.

Our results show that the mutation of Y582 and F629 resulted in almost complete loss of CeDGAT1 function. This result is consistent with an earlier study, showing that replacement of Y392 and F439 of TmDGAT1 (the corresponding residues are Y582 and F629, respectively, in CeDGAT1, Figure 1 and Figure S1) completely abolished DGAT1 activity [21]. This finding suggests that the function of these two conserved sites is essential for different DGAT1s. Numerous substitutions in BnDGAT1 generated by random error PCR have been reported to cause an increase or decrease in activity [27], of which R388 and Q450 variants (the corresponding residues are R596 and Q658, respectively, in CeDGAT1, Figure 1 and Figure S1) with multiple mutations (C286G/F302L/R388S and V52I/E201V/S262T/Q450H, respectively) resulted in increased activity. However, the influence of single sites (R388 and Q450 variants) in BnDGAT1 on function could not be determined. The single amino acid changes in our study further indicate that both mutations decrease DGAT1 function. Moreover, we found another five residues to be essential and seven may have significant effects to varying degrees on the function of CeDGAT1.

Our results showed that Y553A enhanced the activity of CeDGAT1. However, the function of the Y553 site in CeDGAT1 is not similar to that of the corresponding site in other DGAT1s and is specific for CeDGAT1 (Figure 5). The functional difference of Y553 may result from the Y553 position difference in senior structure of different plant DGAT1s, which needs further investigation. It would be valuable to investigate the function of the amino acids of the other plant DGAT1s positioned where Y553 is located in CeDGAT1. Further study is needed to determine whether the other newly identified residues vital for CeDGAT1 function in a similar manner in DGAT1s from other plants.

## 4. Materials and Methods

### 4.1. Yeast Strains and Plasmids

pYES2.0 (Invitrogen, Paisley, UK) was used for constructing yeast expression vectors of *CeDGAT1* and its mutants for expression in *S. cerevisiae* INVSc1 (a His, Leu, Trp and Ura auxotrophic strain) in this study. Yeast mutant H1246 (lacking the four genes *DGA1*, *LRO1*, *ARE1*, and *ARE2* encoding DGAT, PDAT, ASAT1, and ASAT2) was also used for the functional characterization of certain *CeDGAT1* mutant genes.

### 4.2. CeDGAT1 Site-Directed Mutagenesis and Yeast Transformation

The pYES2.0 vector with the inserted *C. ellipsoidea DGAT1* gene *CeDGAT1* [30] (Figure S3) was used as a template to create mutants. Site-directed mutagenesis of *CeDGAT1* was performed using a kit (Beyotime, Shanghai, China) with the primers listed in Table 1.

PCR amplification products of the *CeDGAT1* mutated sequences were digested with *Dpn* I and cloned under the control of the galactose-inducible GAL1 promoter in pYES2.0. Plasmids carrying mutated *CeDGAT1* were verified by sequencing (Sangon, Shanghai, China) and transformed into *S. cerevisiae* INVSc1 and yeast mutant H1246 using the LiAc method [35].

Similarly, we cloned *GmDGAT1* (accession no. AY496439.1), *AtDGAT1* (accession no. NM_127503.2), *BnDGAT1* (accession no. JN224473), and *BoDGAT1* (accession no. XM_013751131.1) cDNAs from *Glycine max*, *A. thaliana*, *B. napus*, and *Brassica oleracea*, respectively, according to published sequences, and mutated these sequences respectively, with respect to amino acid 553 of CeDGAT1 to generate *GmDGAT1Y341A*, *AtDGAT1Y364A*, *BnDGAT1Y347A*, and *BoDGAT1Y352A* using a site-directed mutagenesis kit (Beyotime, Shanghai, China). Yeast expression vectors *GmDGAT1*, *AtDGAT1*, *BnDGAT1*, and *BoDGAT1* and their respective mutants *GmDGAT1Y341A*, *AtDGAT1Y364A, BnDGAT1Y347A*, and *BoDGAT1Y352A* were constructed using the pYES2.0 vector. These genes took the place of CeDGAT1 in Figure S3.

### 4.3. RT-PCR Analysis of Yeast Transformed with CeDGAT1 and Mutants

RNA from yeast-carrying *DGAT1* genes and mutants was extracted using a yeast RNA extraction kit (Kangwee Century, Beijing, China), and cDNA was prepared from 5 μg total RNA template using ReverTra Ace qPCR RT Kit (Toyobo, Osaka, Japan). Yeast *actin* was selected as the reference gene, and expression of *CeDGAT1* mutants was analyzed by RT-PCR using the primer pairs CeDGAT1yeast-f (5′AGTCGGTTCTGGGTGTTCA3′), CeDGAT1yeast-r (5′GCCTGAGTCGGAAGCATAGT3′), Actinyeast-f (5′ACGTCGCCTTGGACTTCGAA3′), and Actinyeast-r (5′AGATGGAGCCAAAGCGGTGA3′). The 25 μL final reaction volume used for PCR contained 2.5 μL of 10 × PCR buffer, 1 μL of each primer (10 μM), 2.0 μL of 2.5 mM dNTPs, 1 μL of cDNA sample, 0.5 μL of Easy-Pfu DNA polymerase (TransGen Biotech, Beijing, China), and 17 μL of double-distilled water. The reaction conditions for PCR were as follow: Denaturation at 95 °C for 10 min, followed by 30 cycles of 94 °C for 30 s, 60 °C for 30 s, and 72 °C for 20 s; and a final extension step of 72 °C for 10 min. The amplified cDNA was cloned into the pEASY-Blunt vector (TransGen Biotech, Beijing, China), and the corresponding clones were verified by PCR and DNA sequencing.

### 4.4. Lipid Analysis by GC–MS and TLC

Total fatty acids of yeast were assessed through GC–MS (gas chromatography–mass spectrometry, TurboMass, PerkinElmer, Waltham, MA, USA), using a previously described protocol [30].

TAG was separated from total lipids through thin-layer chromatography (TLC) on Silica gel 60 plates (Merck, Darmstadt, Germany), using a previously described protocol [30]. The lipids were visualized by spraying primuline (Sigma, Saint Louis, Missouri, USA, 10 mg/100 mL acetone:water (60:40 *v*/*v*)) onto the plates and exposing the plates to UV.

### 4.5. Alignment and Three-Dimensional Structure and Topology Model Prediction

Multiple alignments were performed using MAFFT v6.847b (Osaka, Japan) [22]. The three-dimensional structures and topology model of CeDGAT1 protein were predicted by I-TASSER (Ann Arbor, Michigan, USA) [30,36] and by Protter (Bern, Switzerland) [32] and TMHMM (Pittsburgh, Pennsylvania state, USA) [33], respectively.

### 4.6. Statistical Analysis

Statistical analysis was carried out using Student’s *t* test with the software Statistical Product and Service Solutions (SPSS) (Armonk, New York, NY, USA). The content of TAG was evaluated by semiquantitative densitometry calculated using ImageJ 1.51w (Bethesda, MA, USA).

## 5. Conclusions

Several novel amino acids of CeDGAT1 that are essential and/or important for CeDGAT1 function were identified in this study. Of these, L482, R579, H603, A651, and P660 are essential for CeDGAT1 function, and residues H609, E542, G577, R596, A624, S632, and Q658 are also important. Furthermore, the substitution of residue Y553 markedly increased the total fatty acid content of transgenic yeast, indicating that the highly active *CeDGAT1Y553A* mutant gene may be used to increase the oil content of microalgae or plants. The results obtained in this study provide new information on the structure–function relationships of membrane-bound DGAT1, leading to a better understanding of how the structures of these amino acids influence the function of DGAT1s.

## Figures and Tables

**Table 1:** The mutant names and the primers used for mutagenesis.

Mutants	Primers: Mutagenic Oligonucleotide Sequence (5′-3′, Sense)
L482R	TGGTGGTGCCGGCC*CGG*GTGTACCAGACCAC
E542R	TGGCGGGCATTGTG*CGG*CGCTTGTTGAAGCT
Y553A	CGTTCCTACTTTG*GCC*GGCTGGCTCATCA
G577R	GATCACGTACTTC*CGG*GACCGCGAGTTCT
R579D	GTACTTCGGGGAC*GAC*GAGTTCTACAAGG
Y582R	GGACCGCGAGTTC*CGC*AAGGACTGGTGGA
R596D	TGGGGACTACTGG*GAG*CTGTGGAATGTGC
H603D	GAATGTGCCGGTC*GAC*AAGTGGCTGCTGC
H609D	AGTGGCTGCTGCGG*GAT*GTGTACTACCCCTG
A624R	TCCGAAGACGGTT*CGG*GCAGTGGCGGTGT
F629R	GGCAGTGGCGGTG*CGC*TTCGTGAGTGCCG
S632A	GGTGTTCTTCGTG*GCT*GCCGTGTTCCACG
W650R	CATGGTGCGCTTC*CGG*GCATTCTGGGGCC
A651R	GGTGCGCTTCTGG*CGA*TTCTGGGGCCTCA
Q658H	GGGCCTCATGTTC*CAC*GTGCCTCTGCTGT
P660R	CATGTTCCAGGTG*CGT*CTGCTGTTCTTGA

Note: Codons for the altered amino acids are underlined with black.

## Supplementary Materials

Supplementary materials can be found at http://www.mdpi.com/1422-0067/19/11/3462/s1.

## Abbreviations

DGAT	Diacylglycerol acyltransferase
TAG	Triacylglycerol
TMD	Transmembrane domain
ER	Endoplasmic reticulum
