# Supplementary material for: Newly Identified Essential Amino Acids Affecting Chlorella ellipsoidea DGAT1 Function Revealed by Site-Directed Mutagenesis
Source: Int J Mol Sci. 2018 Nov 4;19(11):3462. doi: 10.3390/ijms19113462 (PMC6274981; doi:10.3390/ijms19113462)
Supplement: Supplementary file 1 [file ijms-19-03462-s001.pdf]

SupplementaryMaterial: Newly Identified Essential Amino Acids Affecting *Chlorella ellipsoidea* DGAT1 Function Revealed by Site-directed Mutagenesis

Baocheng Sun[1, 2*], Xuejie Guo[1*], Chengming Fan[1], Yuhong Chen[1], Jingqiao Wang[3], Zanmin Hu[1, 2**]**

[1] State Key Laboratory of Plant Cell and Chromosome Engineering, Institute of Genetics and Developmental Biology, Chinese Academy of Sciences, Beijing 100101, China; bcsun@genetics.ac.cn
[2] College of Life Sciences, University of Chinese Academy of Sciences, Beijing 100049, China
[3] Institute of Economical Crops, Yunnan Agricultural Academy, Kunming 65023, China
* These authors equally contribute to the research
** Correspondence: cmfan@genetics.ac.cn, Tel.: +86-10-6480-7608; zmhu@genetics.ac.cn, Tel.: +86-10-6480-7626

Table S1. ***DGAT1*** **genes used for predicted protein sequence alignment**

Name	Organism	GenBank No.
CeDGAT1	*Chlorella elliptica*	KT779429
AtDGAT1	*Arabidopsis thaliana*	NP_179535.1
BnDGAT1	*Brassica napus*	JN224473
BoDGAT1	*Brassica oleracea*	XM_013751131.1
BtDGAT1	*Bos Taurus*	AAL49962.1
EaDGAT1	*Euonymus alatus*	AAV31083.1
EpDGAT1	*Echium pitardii*	ACO55634.1
GmDGAT1a	*Glycine max*	AAS78662.1
GmDGAT1b	*Glycine max*	BAE93461.1
HaDGAT1	*Helianthus annuus*	ACD67882.1
HsDGAT1	*Homo sapiens*	NP_036211.2
JcDGAT1	*Jatropha curcas*	ABB84383.1
LjDGAT1	*Lotus japonicas*	AAW51456.1
MmDGAT1	*Mus musculus*	NP_034176.1
MtDGAT1	*Medicago truncatula*	XP_003595231.1
NtDGAT1	*Nicotiana tabacum*	AAF19345.1
OeDGAT1	*Olea europaea*	AAS01606.1
PfDGAT1	*Perilla frutescens*	AAG23696.1
PtDGAT1	*Phaeodactylum tricornutum*	ADY76581.1
RcDGAT1	*Ricinus communis*	NP_001310663.1
SsDGAT1	*Sus scrofa*	NP_999216.1
TgDGAT1	*Toxoplasma gondii*	AAP94209.1
TmDGAT1	*Tropaeolum majus*	AAM03340.2
TpDGAT1	*Thalassiosira pseudonana*	XP_002287215.1
VfDGAT1	*Vernicia fordii*	ABC94471.1
VgDGAT1	*Vernonia galamensis*	ABV21945.1
VvDGAT1	*Vitis vinifera*	XP_002279345.1
ZmDGAT1	*Zea mays*	ABV91586.1

Note: 28 sequences of DGAT1s from 27 different organisms are included in this table

```
CeDGAT1    MPDDASMSQRVEYEGEAQPDRTHVAKCGYLFKYRPYKFTKTWDLRYFELKGKVLEYYLSQKQGANHPRGLVKIEGCVVEI    80
AtDGAT1    ................................................................................     0
BnDGAT1    ................................................................................     0
BoDGAT1    ................................................................................     0
BtDGAT1    ................................................................................     0
EaDGAT1    ................................................................................     0
EpDGAT1    ................................................................................     0
GmDGAT1a   ................................................................................     0
GmDGAT1b   ................................................................................     0
HaDGAT1    ................................................................................     0
HsDGAT1    ................................................................................     0
JcDGAT1    ................................................................................     0
LjDGAT1    ................................................................................     0
MmDGAT1    ................................................................................     0
MtDGAT1    ................................................................................     0
NtDGAT1    ................................................................................     0
OeDGAT1    ................................................................................     0
PfDGAT1    ................................................................................     0
PtDGAT1    ................................................................................     0
RcDGAT1    ................................................................................     0
SsDGAT1    ................................................................................     0
TgDGAT1    ................................................................................     0
TmDGAT1    ................................................................................     0
TpDGAT1    ................................................................................     0
VfDGAT1    ................................................................................     0
VgDGAT1    ................................................................................     0
VvDGAT1    ................................................................................     0
ZmDGAT1    ................................................................................     0

CeDGAT1    EGRKKSRFWVFSVVDPSGLNLLRMSSESEKEVETWVQALVEAGCTKRYLKECFRARSPLRSADIRWKGEDSAG.LKRVPS   159
AtDGAT1    ............................MAILDS....AGVTTVTENGGGEF......VDLDRLRRRKSRS.......DS    35
BnDGAT1    ............................MEILDS....GGVTMPTENGG.........ADLDTLRHRKPRS.......DS    32
BoDGAT1    ............................MAILDS....GGVAVPTENGV.........ADLDRLHRRKSRS.......DS    32
BtDGAT1    ................................................................................     0
EaDGAT1    ..........................................MAANLNEASDLN.......FSLRRR.TGG.......IS    23
EpDGAT1    ............................MTIWES....PEIISS.............DEAAAALRRR.............    22
GmDGAT1a   ............................MAISDE....PETVATALNHS.............SLRRRP..........TA    25
GmDGAT1b   ............................MAISDE....PESVATALNHS.............SLRRRPSAT.......ST    28
HaDGAT1    ...........................................................MSSDVRRR............P     9
HsDGAT1    ................................................................................     0
JcDGAT1    ............................MTILET....TTSGGDGVAESSSDLN.......VSLRRRRKGT.......SS    34
LjDGAT1    ............................MAISED....SESLFAAAAASSVIQSG......SSVRRRP..........SA    32
MmDGAT1    ................................................................................     0
MtDGAT1    ............................MAISDT....PETTATATATVTTIETD.TDLKRSSLRRRPSAT.......ST    40
NtDGAT1    ............................MVIMEL....PESVEMTTTTTTSGIENLNSDLNHSVRRRR...........G    37
OeDGAT1    ............................MTIPEL....PESLETTTLNSHH......SRAASTVRRRSIDVAV...LESD    39
PfDGAT1    ............................MAILDS....PEILDTTSSSADN.....GAAHHTTLRRRQSARSVPPLLDSD    43
PtDGAT1    ............................MDETEITPLLRFSTPSRAEHSSWIKLASESCAYSETDEFLADEAA.RATQRA    51
RcDGAT1    ............................MTILET....PETLGVISSSATSDLN.......LSLRRRRTSN.......DS    34
SsDGAT1    ................................................................................     0
TgDGAT1    ............................MSVVESKRKENGGSGPVTPDIGG..........TSLLKRESK.......VAK    35
TmDGAT1    ............................MAVAES....SQNTTTMSGHGD........SDLNNFRRRKPSSSV...IEPS    37
TpDGAT1    ................................................................................     0
VfDGAT1    ............................MTIPETPDNSTDATTSGGAESSSDLN.......LSLRRRRTAS.......NS    38
VgDGAT1    ............................MALLDT....PQIGEIT............TTATTTIRRRTTVK........P    28
VvDGAT1    ............................MAICNS....PVSVTTSSSSSHADSDL.D....FSIRKRFGGK.......GK    36
ZmDGAT1    ............................MAPPPS...MPAASDRAGPGRDAG.......DSSSLRLRRAPS.......AD    35

CeDGAT1    NQSVLDHRWAGSEESAVTGLETADSLADTLPERDGHARRPRQPLPKDYASDSGMSESGTTAG......PMPRSMTKR...   230
AtDGAT1    SNGLLLSGSDNNSPSDDVG................APADVRDRIDSVVNDDAQGTANLAGDNNGGGDNNGGGRGGGE..G    97
BnDGAT1    SNGLLP....DSVTVSDA....................DVRDRVDSAVEDT.QGKANLAG.......ENEIRESGGE..A    78
BoDGAT1    SNGLLS....DTSPSDDVGA..............AAAEDARDRVDSAVEEEAQGTANLAGG......DTETRESGG...R    85
BtDGAT1    ...MGDRGGAGGSRRRRTGSRPSIQGGSGPAAAEEEVR......DVGAGGDAPVRD...............TDKDGD...    53
EaDGAT1    STTVPD...SSSETSSSE.......ADYLDGG.....KGAADVKDRGDGAVEFQNSMKNVER......IEKHESRVG...    79
EpDGAT1    ..................................GGAKEVAEQR...LDSEEEKKEEEN.....................    45
GmDGAT1a   AGLFNS...PETTTDSSG...............DDLAKDSGS..DDSISSD..AANSQPQ.........QKQDTDFS...    71
GmDGAT1b   AGLFNS...PETTTDSSG...............DDLAKDSGS..DDSINSDDAAVNSQQQN........EKQDTDFS...    77
HaDGAT1    ITNAAD...GTSDSSSSPSP....DVDFNSND............QIAVAHR.....KPNGE............NDRR...    50
HsDGAT1    ...MGDR...GSSRRRRTGSRPSSHGGGGPAAAEEEVRDAAAGPDVGAAGDAPAPA...............PNKDGD...    56
JcDGAT1    DGALPE...LTSNIVELE.........SESGG.QVMMDP.GMVTEPETEKINGKDCGGDKDK......IDNRENRGR...    91
LjDGAT1    ISAVAT...VEDESSSEE...............PVPVRDSGSDVDDSVSSEQHVSPATANR.......EKNQVHDIS...    84
MmDGAT1    ...MGDRGGAGSSRRRRTGSRVSVQGGSGPKVEEDEVRDAAVSPDLGAGGDAPAPAPAPAH.........TRDKDGR...    65
MtDGAT1    AGGLFD...AESAAADAVRDSGSDDSLNGKINNEEEVKDRKTDHAEGIVDDDDDNAVKKNG......GNDVINDRE...   107
NtDGAT1    SNGFEA...ASAINSSDANM....SEDRRDVCGSGAGLETVNERSKSVGESSDVIRKEDDRNDNVANGEESKSTETT...   107
OeDGAT1    SNSLEA...V...NDSDSDV....NNTNEMGNLRGGVVESALEEPSELGTEGLRNGKEENEH...VRTGESNQEMEV...   103
PfDGAT1    SNSLEA...ESAINDSENVR....NDANLIENLRGGAVESENEKQESYGKEEGAKVKENGE........TSNGNGTD...   105
PtDGAT1    LQHQEALQMAQAMPGAKPGTLPPLYFAPTIKRSRSFAKLQEHHGDGMPRVNMRRTKSRDFN.........ADKLDAR...   119
RcDGAT1    DGALAD...LASKFDDDDD......VRSEDSAENIIEDPVAAVTELATAKSNGKDCVANSNK......DKIDSHGGS...    96
SsDGAT1    ...MGDRSGAGGSRRRRTGSRPSSQSGSGFAAAEEEVR......DVGAGGDAPTPD...............KDKDGH...    53
TgDGAT1    SLWLRQQDTITRLEELQR................QHEEEIRAKERRQNSYDDLRTPLRSVDG.......SRRPSVVN...    89
TmDGAT1    SSGFTS...TNGVPATGH..................VAENRDQDRVGAMENATGSVNLIG.......NGGGVVIGNEEKQ    89
TpDGAT1    ...........................................EDPLGRSDIRIS.........................    12
VfDGAT1    DGAVAE...LASKIDELE.........SDAGGGQVIKDP.GAEMDSGTLKSNGKDCGTVKDR......IENRENRGG...    96
VgDGAT1    DAGIGD...GLFDSSSSSKT....NSSFEDGDSLNGDFNDKFKEQIGAGDESKDDSKGNGQK....IDHGGVKKGRE...    94
VvDGAT1    AVADSS...LETETEAAA...............AAVLEAEKSVGEVGSGGDRGESGSQVVRN......GENGVAEVA...    89
ZmDGAT1    AGDLAG...............................DSSGGLRENGEPQSPTNPPPQEQQQ..................    66
```

```
                                               I
CeDGAT1    .....HSDMRGSTQLYTESRPSILSTERFAFTQ.HSGILNLMTMVLLATNARLIIENLMKYGVLTNPTRWLLFLVPKGHT   304
AtDGAT1    RGNADATFTYRPSVPAHRRARESPLSSDAIFKQSHAGLFNLCVVVLIAVNSRLIIENLMKYGWLIRTD...FWFSSRSLR   174
BnDGAT1    GGNVDVRYTYRPSVPAHRRVRESPLSSDAIFKQSHAGLFNLCVVVLVAVNSRLIIENLMKYGWLIRTD...FWFSSTSLR   155
BoDGAT1    GGNGDVRFTYRPSVPAHRRTRESPLSSDAIFKQSHAGLFNLCVVVLVAVNSRLIIENLMKYGWLIRTD...FWFSSTSLR   162
BtDGAT1    .....VDVGSGHWDLRCHRLQDSLFSSDSGFSN.YRGILNWCVVMLILSNARLFLENLIKYGILVDPIQVVSLFLKDPYS   127
EaDGAT1    L...DSRFTYRPSVPAHRTIKESPLSSDAIFKQSHAGLFNLCIVVLVAVNSRLIIENLMKYGWLIRSG...FWFSSRSLR   153
EpDGAT1    ...GKLKYTYRASAPAHRRIKESPLSSDAIFKQSHAGLFNLCIVVLVAVNSRLIIENLMKYGWLISSG...FWLSSTSLS   119
GmDGAT1a   ....VLKFAYRPSVPAHRKVKESPLSSDTIFRQSHAGLFNLCIVVLVAVNSRLIIENLMKYGWLIKSG...FWFSSKSLR   144
GmDGAT1b   ....VLKFAYRPSVPAHRKVKESPLSSDTIFRQSHAGLFNLCIVVLVAVNSRLIIENLMKYGWLIKSG...FWFSSKSLR   150
HaDGAT1    R..SVMHYAYRPCTPAHNRIKESPLSSDAIFKQSHAGLFNLCIVVLVAVNSRLIIENLMKYGLLINAN...FWFSSRSLR   125
HsDGAT1    .....AGVGSGHWELRCHRLQDSLFSSDSGFSN.YRGILNWCVVMLILSNARLFLENLIKYGILVDPIQVVSLFLKDPYS   130
JcDGAT1    S...DIKFTYRPSVPAHRALRESPLSSDAIFKQSHAGLFNLCIVVLVAVNSRLIIENLMKYGWLIKTG...FWFSSRSLR   165
LjDGAT1    ....ATKFAYRPSAPAHRRVKESPLSSDNIFRH.HAGLFNLCIVVLVAVNSRLIIENLMKYGWLIRTG...FWFSSKSLR   156
MmDGAT1    .....TSVGDGYWDLRCHRLQDSLFSSDSGFSN.YRGILNWCVVMLILSNARLFLENLIKYGILVDPIQVVSLFLKDPYS   139
MtDGAT1    NVAVDFKFTYRPSVPAHRRSKESPLSSGNIFRQSHAGLFNLCIVVLVAVNSRLIIENLMKYGWLIRSG...FWFSSKSLR   184
NtDGAT1    T..TPFKFAYRASAPAHRRIKESPLSSDAIFKQSHAGLFNLCVVVLIAVNSRLIIENLMKYGLLIRAG...FWFSSKSLR   182
OeDGAT1    L..ASAKFAHRPSAPVHRRIKESPLSSDAIFKQSHAGLFNLCIVVLVAVNSRLIIENLMKYGWLINSG...FWFSSTSLK   178
PfDGAT1    V..MAVKFTFRPAAPAHRKNKESPLSSDAIFKQSHAGLFNLCIVVLVAVNSRLIIENLMKYGWLIKSG...FWFSSTSLR   180
PtDGAT1    .....STKGYPPSKPMHRAAEPSYLSADAPIQN.YRGFLNLGVIILIVSNFRLILGTIRSNGFVLTTAVKHYKNLNHLKE   193
RcDGAT1    S...DFKLAYRPSVPAHRSLKESPLSSDLIFKQSHAGLFNLCIVVLVAVNSRLIIENLMKYGWLIKTG...FWFSSRSLR   170
SsDGAT1    .....DDVSSGHWDLRCHRLQDSLFSSDSGFSN.YRGILNWCVVMLVLSNARLFLENLIKYGILVDPIQVVSLFLKDPYS   127
TgDGAT1    ...DINDYRGRPLHDQLHGAYRATLLSHHTKNLNLRGFLNLFFILLFVINFRMATDNLMEYGLMIRLP...SSFRDILSN   163
TmDGAT1    VGETDIRFTYRPSFPAHRRVRESPLSSDAIFKQSHAGLFNLCIVVLIAVNSRLIIENLMKYGWLIDTG...FWFSSRSLG   166
TpDGAT1    .........YPPSKPMHRQSNPSYLSDGSHVQN.YRGLFNLLLLILVLSNFRLLLDTVAQHGFILD....KLATLQGFSQ    78
VfDGAT1    S...DVKFTYRPSVPAHRALKESPLSSDNIFKQSHAGLFNLCIVVLVAVNSRLIIENIMKYGWLIKTG...FWFSSRSLR   170
VgDGAT1    T..TVVHYAYRPSSPAHRRIKESPLSSDAIFKQSHAGLFNLCIVVLVAVNGRLIIENLMKYGLLINSN...FWFSSRSLR   169
VvDGAT1    .....AKFAYRPCAPAHRKVKESPLSSDAIFRQSHAGLFNLCIVVLVAVNSRLIIENLMKYGWLIRAG...FWFSSKSLR   161
ZmDGAT1    ....HEMLYYRASAPAHRRVKESPLSSDAIFRQSHAGLLNLCIVVLIAVNSRLIIENLMKYGLLIRAG...FWFSARSLG   139

CeDGAT1    NLMYCWPAMALFALIALGIEKLGAKRLALERKASMAKRKRDMRPVEARRKAAQMAARSECLLLSLHLANLSAVLVLPTWV   384
AtDGAT1    ....DWPLFMCCISLSI....FPLAAFTVEKLVLQK......................YISEPVVIFLHIIITMTEVLYPVYV   227
BnDGAT1    ....DWPLFMCCLSLSI....FPLAAFTVEKLVLQK......................CISEPVVIILHIIITMTEVLYPVYV   208
BoDGAT1    ....DWPLFMCCLSLSI....FPLAAFTVEKMVLQK......................FISEPVAIILHVIITLTEVLYPVYV   215
BtDGAT1    .....WPALCLVIVANI....FAVAAFQVEKRLAVG......................ALTEQAGLLLHGVNLATILCFPAAV   179
EaDGAT1    ....DWPLFMCCLTLPV....FPLAAFLFEKLAQKN......................LISEPVVVLLHIVNTTAAVLYPVLV   206
EpDGAT1    ....DWPLLICCLSLPI....FPLASFVVEKLSQQE......................FISEQVVITLHALITTTVIMYPVIV   172
GmDGAT1a   ....DWPLFMCCLSLVV....FPFAAFIVEKLAQQK......................CIPEPVVVVLHIIITSASLFYPVLV   197
GmDGAT1b   ....DWPLFMCCLSLVV....FPFAAFIVEKLAQRK......................CIPEPVVVVLHIIITSTSLFYPVLV   203
HaDGAT1    ....DWPLLMCCLSLPV....FPLAAYIVEKFAWTK......................RIADSAAITLHIIITTIAILYPVFM   178
HsDGAT1    .....WPAPCLVIAANV....FAVAAFQVEKRLAVG......................ALTEQAGLLLHVANLATILCFPAAV   182
JcDGAT1    ....DWPLLMCCLTLPI....FSLAAYLVEKLAYRK......................YISAPIVIFFHMLITTTAVLYPVSV   218
LjDGAT1    ....DWPLFMCCLSLAI....FPFAAFVVEKLVQQK......................CISEP.VVVLHIFISTAAVVYPVLV   208
MmDGAT1    .....WPAPCVIIASNI....FVVAAFQIEKRLAVG......................ALTEQMGLLLHVVNLATIICFPAAV   191
MtDGAT1    ....DWPLFMCCLSLAI....FPLAAFVVEKLAQQK......................RISEPVIVLLHIVITTVISYGSMAL   237
NtDGAT1    ....DWPLLMCCLSLQI....LPLAAFLVEKLAQQR......................HLTERAVVTLHITITTAAILYPVLV   235
OeDGAT1    ....DWPLLMCCLSLPI....FPLAAFFVEKLVLLK......................YISECVAVFLHILITTAAILYPVLV   231
PfDGAT1    ....DWPLLMCCLSLPV....FALASFLVEKLVKLN......................YIPEWVAVFLHVTITTVEILFPVVV   233
PtDGAT1    DPWQEFPFVSGFLLQLV....FVSIAFGIEWMLCRK......................YFNENFGMILHHFNAHSALLIPLGI   250
RcDGAT1    ....DWPLFMCCLSLPV....FPLAAYLVEKAAYRK......................YISPPIVIFLHVIITSAAVLYPASV   223
SsDGAT1    .....WPALCLVIVANV....FAVTAFQVEKRLAVG......................ALTEQAGLLIHVANLATILCFPAAV   179
TgDGAT1    .....WPMLLCFLIMHF....CILGAFVIERFVAAWVP.................HLLDWSECVLIGLNFFLVFIVPYLT   217
TmDGAT1    ....DWSIFMCCLTLPI....FPLAAFIVEKLVQRN......................HISELVAVLLHVIVSTAAVLYPVIV   219
TpDGAT1    AP.LDFPFVSGLLIVQA....FVVGAYAIEKMLSVG......................LIGNQFGMLLHVINSNATLGVVMAI   134
VfDGAT1    ....DWPLLMCCLTLPI....FSLAAYLVEKLACRK......................YISAPTVVFLHILFSSTAVLYPVSV   223
VgDGAT1    ....DWPLLMCCLTPSD....FPLAAYIVEKLAWKK......................RISDPVVITLHVIITTTAILYPVFM   222
VvDGAT1    ....DWPLFMCCLTLPI....FPLAAFVVEKLAQQK......................YISEQVVVSLHIIITTAAVLFPVLV   214
ZmDGAT1    ....DWPLLMCCLTLPV....FPLVALMAEKLITRK......................LIGEHVVILLHIIITTSAIVYPVVV   192

CeDGAT1    VLISD..TPVPSFMVIIFTITLWMKLTSYAHCNLDFRRLSR....DKERRRADRPSRPGGEGLGEMDIPETIHQGVEAVP   458
AtDGAT1    TLRCDS.AFLSGVTLMLLTCIVWLKLVSYAHTSYDIRSLAN.........AADK..ANP.....................   274
BnDGAT1    TLRCDS.AFLSGVTLMLLTCIVWLKLVSYAHTNYDIRTLAN.........SSDK..ANP.....................   255
BoDGAT1    TLRCDS.AFLSGVTLMLLTCIVWLKLVSYAHTSYDIRTLAN.........SADK..VDP.....................   262
BtDGAT1    AFLLESITPVGSVLALMVYTILFLKLFSYRDVNLWCRERRA....GAKAKAALAGKKANG.................GAA   238
EaDGAT1    ILRCDS.AFMSGVTLMLFACIVWLKLVSYAHTNYDMRALTK.........SVEKGDTPLS...................S   257
EpDGAT1    ILRCDP.AVLSGVILMLFTCIVWLKLVSYAHTNYDMRALAK.........DCDKLQALSG...................S   223
GmDGAT1a   ILRCDS.AFLSGVTLMLFACVVWLKLVSYAHTNYDMRALTK.........SVEKGEALPD...................T   248
GmDGAT1b   ILRCDS.AFVSGVTLMLFSCVVWLKLVSYAHTNYDMRALTK.........LVEKGEALLD...................T   254
HaDGAT1    IMRFDS.AVLSGVTLMLFACVNWLKLVSFVHANYDMRMLVN.........SADKEKEKSA...................S   229
HsDGAT1    VLLVESITPVGSLLALMAHTILFLKLFSYRDVNSWCRR........ARAKAASAGKKASS.................AAA   237
JcDGAT1    ILSCGS.AVLSGVALMLFACIVWLKLVSYAHTNYDMRAIAN.........SADKGDALSD...................T   269
LjDGAT1    ILRTDS.AFPSGVTLMLFACIVWLKLVSYAHTNYDMRELTK.........SIEKGEALPN...................T   259
MmDGAT1    ALLVESITPVGSVFALASYSIMFLKLYSYRDVNLWCRQRR......VKAKAVSTGKKVSG.................AAA   248
MtDGAT1    LCRCDS.AFLSGSTLMLLTCIVWLKLVSYAHTTYDMRALAV.........SNEKGETMPD...................T   288
NtDGAT1    ILGCDS.AFLFGVILMLVACIVWMKLVSYAHTNHDMRQLAK.........STDKDETSDG....................   285
OeDGAT1    ILRCDS.AVLSGVTLMLFACIVWLKLVSYAHASHDMRALAK.........SLDKGETLSG...................Y   282
PfDGAT1    ILRCDS.AVLSGVTLMLFACTVWLKLVSYAHTNYDLRVLAK.........SLDKWEAMSR...................Y   284
PtDGAT1    VWNLID.RPAVGAILLLHATITWMKLISYMLANEDYRLSSRRVGGNPHLATLALVENLDS...................D   310
RcDGAT1    ILSCES.AFLSGVTLMELACMVWLKLVSYAHTNYDMRAIAD.........TIHKEDAS.N...................S   273
SsDGAT1    AFLLESITPVGSLLALMVYAILFLKLFSYRDVNLWCRERRA....TAKAKAASAGKKANG.................GAA   238
TgDGAT1    VCNSSP.SPAASALLLAFSIVWLFKLFSFHHVCLDARRALR.........DGDNFRELCSNS...............EEA   272
TmDGAT1    ILTCDS.VYMSGVVLMLFGCIMWLKLVSYAHTSSDIRTLAK.........SGYKGDAHPN...................S   270
TpDGAT1    VWYLID.QPFVGAGLIMQATITWLKLISYAHANYDYRTSP.....DTQKVTVALVKDLD....................D   188
VfDGAT1    ILSCES.AVLSGVALMLFACIVWLKLVSYAHTNFDMRAIAN.........SVDKGDALSN...................A   274
VgDGAT1    ILRFDS.VVLSGVSLMLCACINWLKLVSFVHTNYDMRSLLN.........STDKGEVEPM...................S   273
VvDGAT1    ILRCDS.AVLSGVTLMLFACIVWLKLVSFAHTNYDMRAVAK.........LIDKGDDLST...................S   265
ZmDGAT1    TLKCDS.AVLSGFVLMLFASIMWMKLVSYAHTNYDIRVLSK.........STEKGAAYGN...................Y   243
```

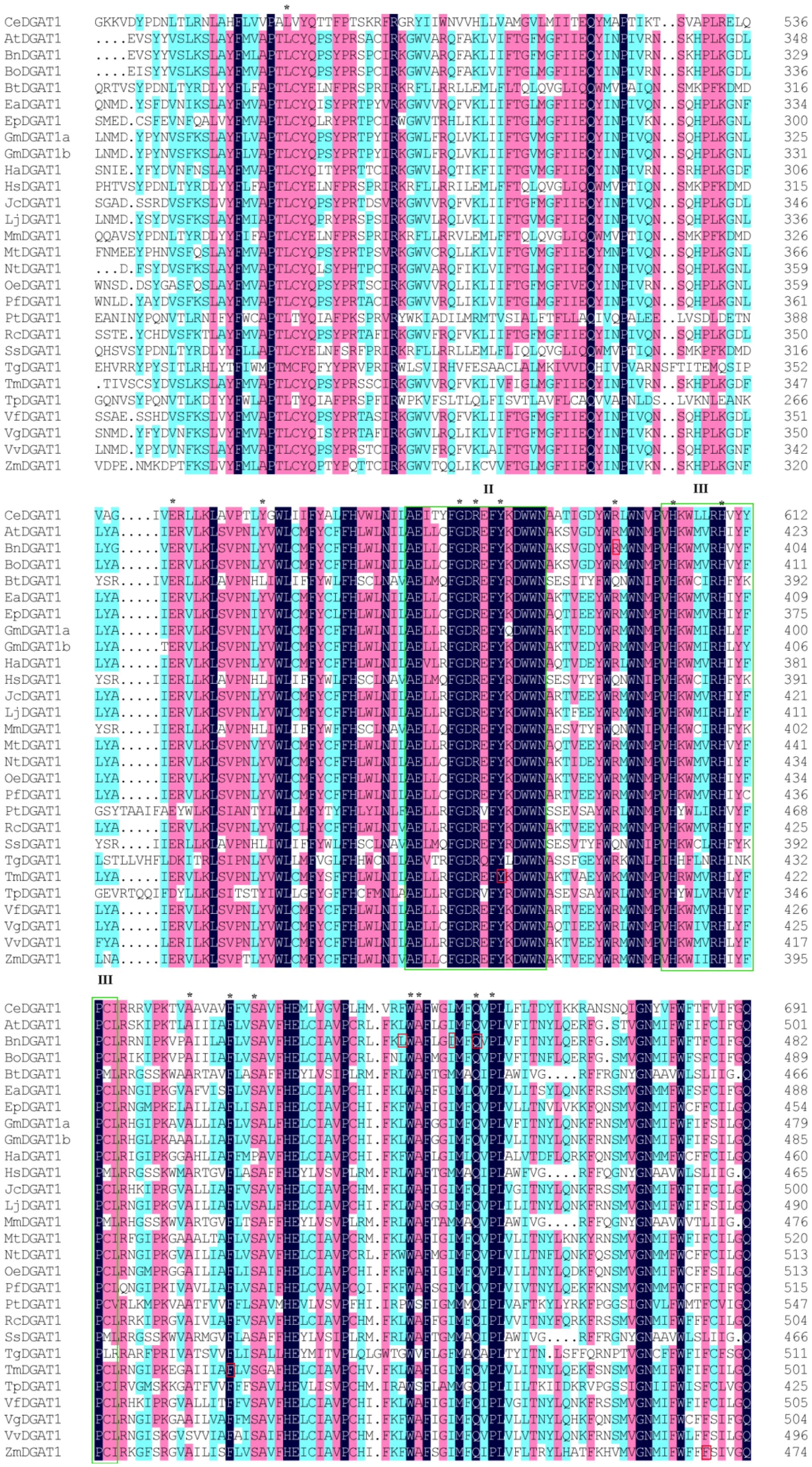
II
III

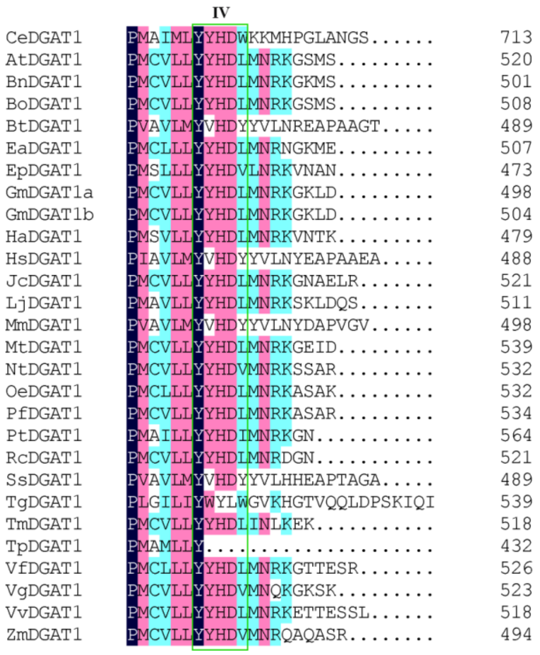

Figure S1. Multiple full length sequence alignment of the 28 DGAT1s from 27 species and mutagenesis site selection The deduced amino acid sequences of DGAT1s were aligned using MAFFT v6.847b [30] with the L-INS-i algorithm. Conserved amino acids are shaded in black (90-100% similarity, the highest conservation), pink (70-90% similarity, higher conservation) and light blue (50-70% similarity, high conservation). Conserved motifs (domains I, II, III and domain IV) are marked by green rectangles, S197, P216, Y392 and F439 in TmDGAT1; F469 in ZmDGAT1; R388, L441, I447 and Q450 in BnDGAT1 are marked by red boxes, which were previously proved affecting the activity of DGAT1 in *Tropaeolum majus*, *Zea mays* and *Brassica napus*, respectively and the 16 selected conserved residues mutated are labelled by asterisks. The GenBank accession numbers and sources of *DGAT1* genes are listed in supplementary file Table S1).

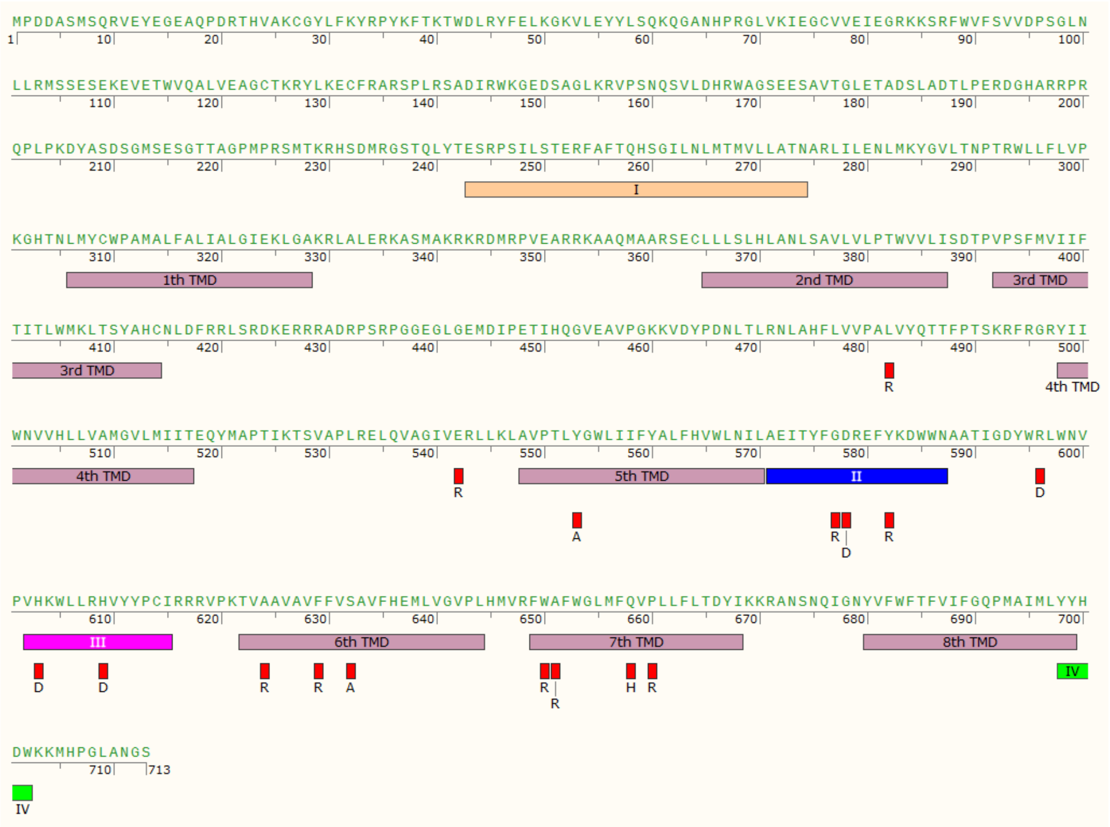

Figure S2. The sequence of the CeDGAT1 protein and single mutation sites

The functional sites predicted by the Prosite database are labelled with different colours: I, the domain of acyl-CoA binding signature; II, the domain of fatty acid protein signature; III, the domain of the DAG-binding site; and IV, the domain of the putative endoplasmic reticulum retrieval motif at the C-terminus. TMD, transmembrane domain. Single mutation sites are marked with red boxes.

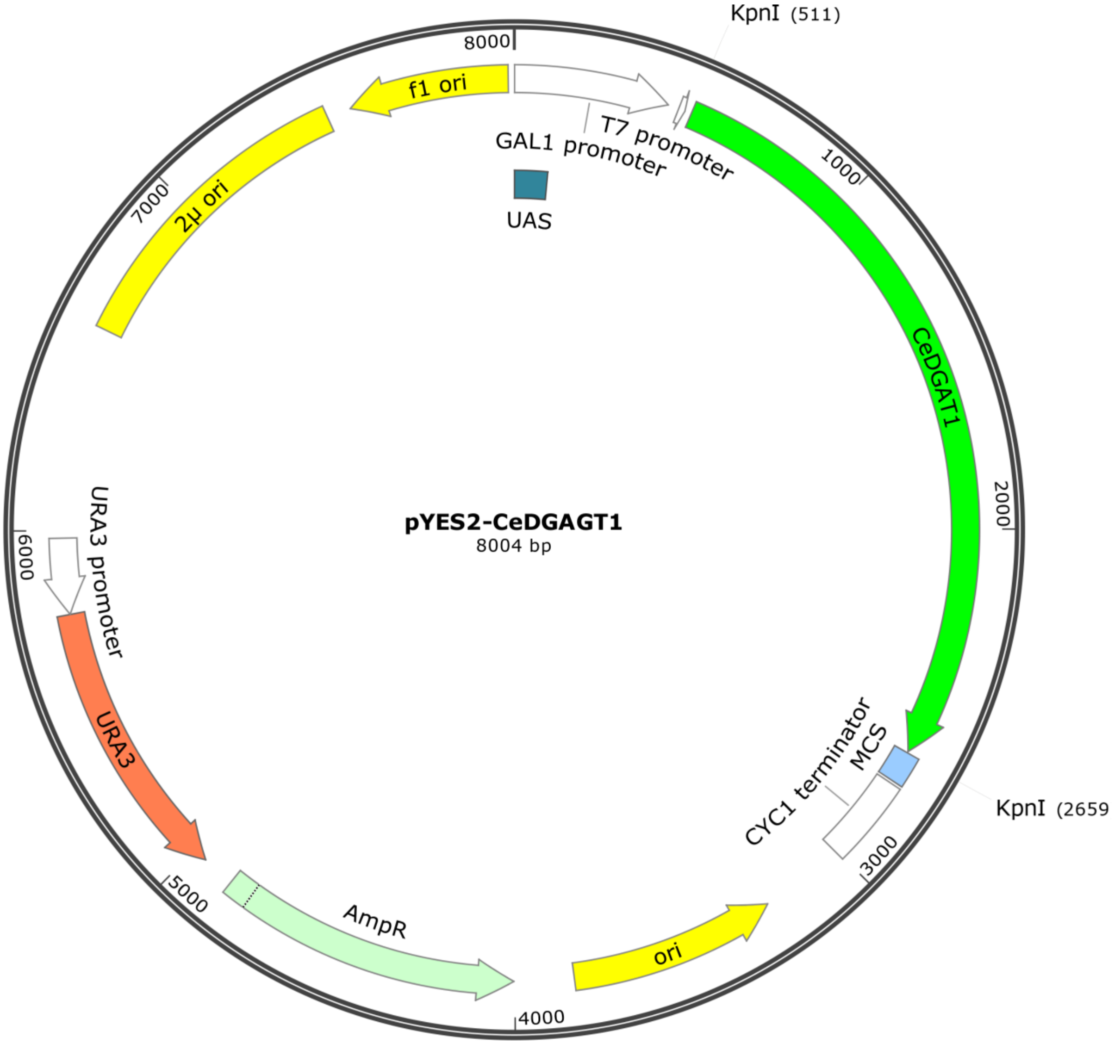

Figure S3. The diagram of the vector pYES2.0 carrying *CeDGAT1* and mutants The mutants were cloned in the same site of pYES2.0 as *CeDGAT1*.

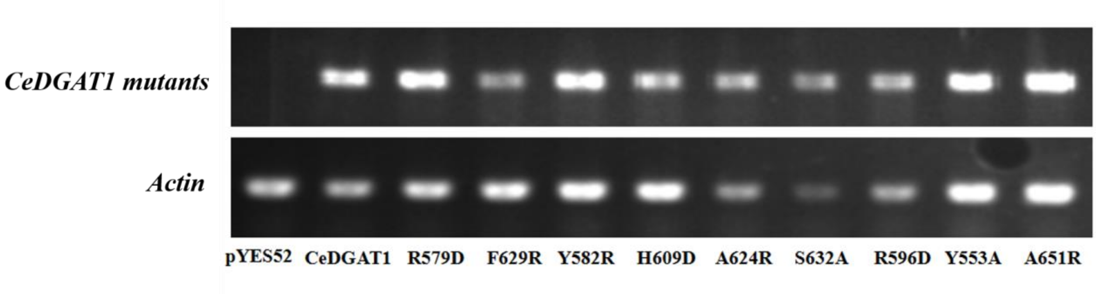

Figure S4. Expression of a portion of *CeDGAT1* mutant genes in transgenic yeast Yeast *actin* was used as an internal control. Yeast cells harbouring the empty pYES2.0 vector were used as the negative control. The lanes are indicated by the name of mutation.

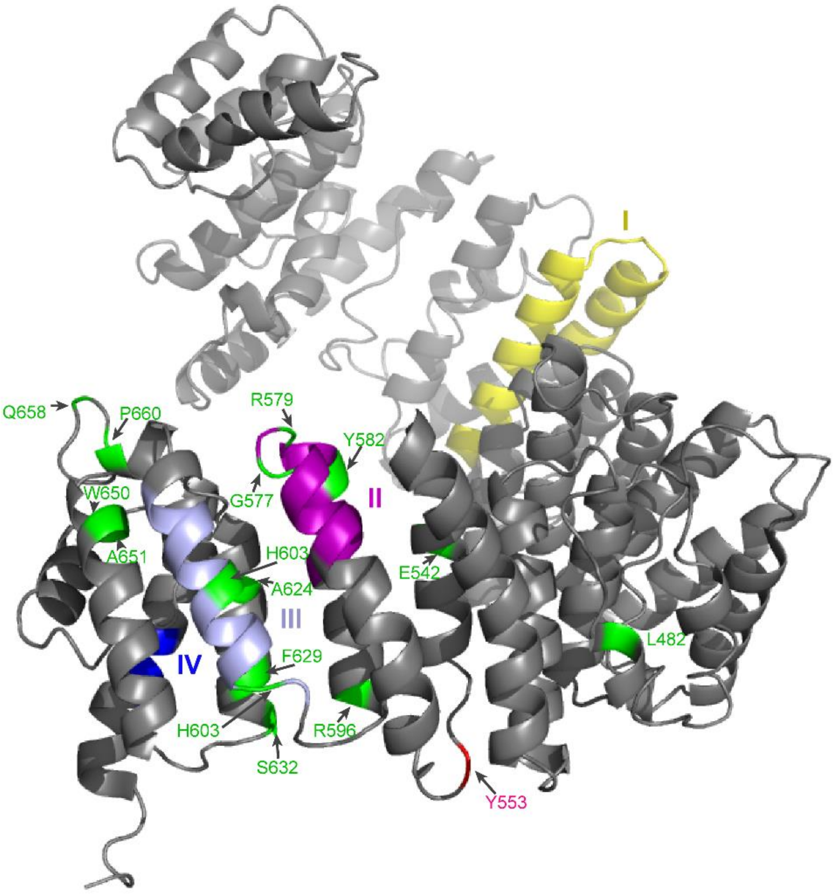

Figure S5. The 16 mutation sites on the predicted three-dimensional structure of CeDGAT1 The three-dimensional structure of CeDGAT1 was predicted by I-TASSER [23]. The mutation sites of CeDGAT1 and the four functional domains of CeDGAT1 are shown in the predicted model. I, from E243 to N274, the acyl-CoA binding signature in yellow; II, from A571 to N587, the domain of fatty acid protein signature which contains a tyrosine phosphorylation site in purple; III, from V602 to I615, the domain of the DAG-binding site in light blue; and IV, from Y698 to W702, the domain of the putative endoplasmic reticulum retrieval motif in the C-terminus in blue. The 15 amino acids are in green except for Y553 in red.
